# HER-2 and HER-3 expression in liver metastases of patients with colorectal cancer

**DOI:** 10.18632/oncotarget.3527

**Published:** 2015-04-13

**Authors:** Hanna Styczen, Iris Nagelmeier, Tim Beissbarth, Manuel Nietert, Kia Homayounfar, Thilo Sprenger, Ute Boczek, Kathrin Stanek, Julia Kitz, Hendrik A. Wolff, B. Michael Ghadimi, Peter Middel, Torsten Liersch, Josef Rüschoff, Lena-Christin Conradi

**Affiliations:** ^1^ Department of General, Visceral and Pediatric Surgery, University Medical Center, Göttingen, Germany; ^2^ Targos Molecular Pathology, Pathology Nordhessen, Kassel, Germany; ^3^ Department of Medical Statistics, University Medical Center, Göttingen, Germany; ^4^ Department of Pathology, University Medical Center, Göttingen, Germany; ^5^ Department of Radiotherapy and Radiooncology, University Medical Center, Göttingen, Germany

**Keywords:** colorectal cancer, HER-2, HER-3, targeted therapy, liver metastases

## Abstract

**Objective:**

In this study, we evaluate the frequency of HER-2 and HER-3 expression in liver metastases from patients with colorectal cancer (CRLM). We analyzed the potential of HER-2 and HER-3 as therapeutic targets and evaluated their prognostic value.

**Patients and Methods:**

Overall 208 patients with CRLM were enrolled. HER-2 and HER-3 expression were determined in metastatic tissue of diagnostic punch biopsies (*n* = 29) or resection specimens (*n* = 179). The results of immunohistochemistry (IHC) scoring and In-situ-hybridization (ISH)-amplification were correlated with clinical parameters and for the 179 resected patients with cancer-specific (CSS) and overall survival (OS). The mean follow-up time was 56.7 months.

**Results:**

Positivity of HER-2 status (IHC score 2+/ISH+ and IHC 3+) was found in 8.2% of CRLM. High expression of HER-3 (IHC score 2+ and IHC 3+) was detected in 75.0% of liver metastases. CSS after liver surgery was determined and was independent from the HER-2 status (*p* = 0.963); however HER-3 was prognostic with a favorable course for patients showing an overexpression of HER-3 (*p* = 0.037).

**Conclusions:**

HER-2 overexpression occurs in only 8% of patients with CRLM but with 75% of cases HER-3 is frequently overexpressed in CRLM. Therefore, HER-2 and particularly HER-3 could serve as novel targets to be addressed within multimodal treatment approaches.

## INTRODUCTION

Colorectal cancer (CRC) is the third most common cancer in the United States and also the third leading cause of cancer related deaths [1]. High incidence rates of CRC are reported mainly in developed countries with a Western culture including the United States, Australia, New Zealand, Canada and Western Europe [2][3][4]. The implementation of multimodal therapy including preoperative chemoradiotherapy (CRT) has led to an reduction of local recurrences, the introduction of total mesorectal excision (TME) followed by adjuvant chemotherapy with 5-FU and oxaliplatin [5] and complete mesocolic excision (CME) as well as the availability of new biological substances in the last two decades are aiming to improve prognosis of patients with (metastatic) CRC [6][7][8].

However, the occurrence of distant metastases limits the prognosis of these patients. Rates up to 30–50% of metastases during the course of malignancy are reported with a predominant location in the liver followed by the lung. Besides Radiofrequency ablation (RFA) and stereotactic body radiation therapy (SBRT) histopathologically confirmed R0 resection in potentially curative intent is a favorable option in case of technical resectability. Due to technical progress in liver surgery and access to innovative treatment approaches, the prognosis of patients with colorectal liver metastases has dramatically improved [9]. Data on survival vary, but about 17–32% of patients qualify for surgical resection of liver metastases [10][11], leading to 5-year overall survival (OS) rates ranging from 32–33% [11][12] to up to 58% [13] and 10-year OS rates of about 18% [11].

For those patients, whose metastases remain surgically unresectable and who are treated with chemotherapy (CTx) alone, median survival rates are currently reported with 13.9 - 17.4 months [14][15]. In the last decades, metastatic CRC has been treated with Fluoropyrimidine-based CTx and recently in the combination with irinotecan or oxaliplatin. Due to non-specificity of this treatment, there have been major initiatives in targeted therapy of CRC using antibody based products such as cetuximab. In several studies including recent data from the CELIM trial, it has been demonstrated that initially unresectable colorectal liver metastases can be resected after response to cetuximab based CTx leading to a better overall survival of these patients [16][17]. Patients who respond to conversion therapy and undergo secondary surgery achieve a better median OS of 53.9 months compared to those who do not (21.9 months; *p* < 0.001) [16]. Still, the evaluation of new agents targeting molecular pathways that are expressed strongly by tumor cells is crucial to develop innovative treatment approaches.

In this context we evaluated the transmembrane receptor HER-2, a family member of the epidermal growth factor receptor family (EGFR). HER-2 is already known as a prognostic biomarker in various solid tumors (for more than a decade in breast cancer [18][19], more recently for gastric cancer [20], esophageal adenocarcinoma [21], pancreatic [22] and rectal cancer [23]) and even more importantly as a potential target in the specific tumor therapy in clinical routine for breast and metastatic gastric cancer. Data on the prognostic value of HER-2 in GI-malignancies still remains less clear. The ToGA-trial showed a HER-2 positivity of 22.1% [20][24] in gastric cancer and adenocarcinoma of the gastro-esophageal junction (overall *n* = 584 HER-2 positive) using a specific modified immunohistochemistry (IHC) scoring algorithm in comparison to breast cancer [20][25]. Patients with overexpression of HER-2 (IHC 3+, IHC 2+/FISH+) had a better survival compared to patients with no or weak HER-2 expression levels (IHC 0, IHC 1+, IHC 2+/FISH-). These patients treated with trastuzumab additionally to standard CTx benefited significantly from the individualized therapy [20]. Comparable studies using the same IHC scoring criteria showed a HER-2 positivity of 17% in adenocarcinomas of the esophagus and of 27% in resection specimens of patients with cUICC-II/-III rectal cancer [21][23]. Both studies demonstrated the correlation of a high HER-2 expression with better survival in gastrointestinal cancer. On the contrary, in breast cancer HER-2 positivity seems to be associated with a higher occurrence of brain metastases and reduced survival.

In several published meta-analyses it has been shown that HER-2 overexpression was correlated with decreased survival in CRC patients suggesting that HER-2 overexpression might not be a prognostic indicator [26][27]. In metastatic CRC, Aprile et al. [28] reported a negative prognostic value of HER-2 expression in brain metastases.

In this study we evaluate the frequency of HER-2 positivity in CRLM and in a very small subgroup of patients also in corresponding primary tumor resection specimens. Furthermore we analyzed if HER-2 could be a prognostic biomarker or could, when being overexpressed, in the future serve as a therapeutic target. In addition, we assessed the expression of the EGFR family member HER-3 whose role in metastatic CRC has not been fully analyzed and is not understood yet. A recent publication showed a HER-3 expression in about 70% of primary tumors of stage II and III CRC and in about 75% of corresponding lymph node metastases [29]. High expression of HER-3 was even assessed with worse clinical outcome. So HER-3 could remain as an independent (from post-resection therapy) prognostic factor for distant metastases [29][30] and could even be targeted specifically by a therapeutic antibody [31].

## RESULTS

### HER-2 status in liver metastases and corresponding primary tumors

Positive HER-2 status (IHC 2+/ISH+ and IHC 3+) was detected in 8.2% (17/208) of CRLM and in 18.2% (4/22) of the very small subgroup of patients with a resection specimens in primary tumors (Table 3). Dual-ISH was performed in 46 specimens (33 metastases and 13 primary tumors) classified IHC 2+, to determine gene amplification. About 75% of tissue specimens revealed heterogeneous or focal expression (≤ 30% of HER-2 positive cancer cells) of HER-2. Positivity of HER-2 status was found in 6% of synchronous metastases and in 11% of the metachronous metastases (Table 1). Furthermore, HER-2 was in trend more often overexpressed in metastases of colon cancer (*n* = 8; 12.1%) compared to metastases of rectal adenocarcinomas (*n* = 9; 6.3%).

**Table 1:** Demographics and clinical parameters (*N* = 208)

Clinical Parameters	HER-2					HER-3		
	*N* = 208	%	Low expression	High expression	*p*-value	Low expression	High expression	*p*-value
**Age, median and range**	67.5 years (40 – 90 years)							
**Gender** Female Male	71 137	34 66	65 (91.5%) 126 (91.9%)	6 (8.5% 11 (8.0%)	NS	12 (16.9%) 40 (29.2%)	59 (83.1%) 97 (70.8%)	NS
**Distant metastases** Hepatic Further distant organ metastases	117 91	56 44	109 (93.2%) 82 (90.1%)	8 (6.8%) 9 (9.9%)	NS	28 (23.9%) 24 (26.4%)	89 (76.1%) 67 (73.6%)	NS
**Hepatic metastases** One lobe of the liver Two lobes Diffuse metastases	105 89 14	50 43 7	96 (91.4%) 82 (92.1%) 13 (92.9%)	9 (8.6%) 7 (7.9%) 1 (7.1%)	NS	22 (21.0%) 24 (27.0%) 6 (42.9%)	83 (79.0%) 65 (73.0%) 8 (57.1%)	NS
**Hepatic metastases** Synchronous Metachronous	118 90	57 43	111 (94.1%) 80 (88.9%)	7 (5.9%) 10 (11.1%)	NS	34 (28.8%) 18 (20.0%)	84 (71.2%) 72 (80.0%)	NS
**Primary tumor** Colon - right sided - left sided Rectum	20 46 142	10 22 68	16(80.0%) 42 (91.3%) 133 (93.7%)	4 (20.0%) 4 (8.7%) 9 (6.3%)	NS	5 (25.0%) 9 (19.6%) 38 (26.8%)	15 (75.0%) 37 (80.4%) 104 (73.2%)	NS
Resected primary tumor Not resected primary tumor	203 5	98 2	186 (91.6%) 5 (100%)	17 (8.4%) 0		51 (25.1%) 1 (20%)	152 (74.9%) 4 (80%)	
**UICC-Status at diagnosis of the primary** I II III IV NA	5 10 18 118 57	2 5 9 57 27	5 (100%) 10 (100%) 15 (83.3%) 111 (94.1%) 50 (87.7%)	0 0 3 (16.7%) 7 (5.9%) 7 (12.3%)	NS	3 (60%) 0 5 (27.8%) 34 (28.8%) 10 (17.5%)	2 (40%) 10 (100%) 13 (72.2%) 84 (71.2%) 47 (82.5%)	NS

Basic clinical data are shown according to HER-2 and HER-3 status of hepatic metastases (low HER-2 expression = IHC 0, IHC 1+, IHC 2+/ISH negative, high HER-2 expression = IHC 3+, IHC 2+/ISH positive; low HER-3 expression = IHC 0, IHC 1+, high HER-3 expression = IHC 2+, IHC 3+)

**Table 2:** Long-term follow up of patients

Follow Up Last update 12/2013	HER-2					HER-3		
	*N* = 208	%	Low Expression	High expression	*p*-value	Low expression	High expression	*p*-value
**Mean and Range**	56.7 months (0.6 - 277.8 months)							
**Cancer related death**	134	64	125 (93.3%)	9 (6.7%)		36 (26.9%)	98 (73.1%)	
**No cancer related death**	14	7	14 (100%)	0	NS	3 (21.4%)	11 (78.6%)	NS
**Still alive**	60	29	52 (86.7%)	8 (13.3%)		13 (21.7%)	47 (78.3%)	

**Table 3:** HER-2 expression in metastases and primary tumors assessed with IHC and Dual-ISH and HER-3 expression as assessed by IHC

IHC/ISH score for HER-2 expression	0	1+	2- (ISH-)	2+ (ISH+)	3+
HER-2 expression and gene amplification in metastases (*n* = 208)	133 (63.9%)	33 (15.9%)	25 (12.0%)	8 (3.8%)	9 (4.3%)
HER-2 expression and gene amplification in primary tumors (*n* = 22)	3 (13.6%)	5 (22.7%)	10 (45.5%)	3 (13.6%)	1 (4.5%)
**IHC score for HER-3 expression**	**0**	**1+**	**2+**		**3+**
HER-3 expression in metastases (*n* = 208)	51 (24.5%)	1 (0.5%)	63 (30.3%)		93 (44.7%)
HER-3 expression in primary tumors (*n* = 22)	5 (22.7%)	1 (4.5%)	2 (9.1%)		14 (63.6%)

Within the 22 matched paired samples, 4 primary cancer samples and 2 CRLM samples showed HER-2 overexpression but in none of these cases the matched tissue samples showed this overexpression in the same way (Table 4 *p* = 0.48).

**Table 4:** HER-2 and HER-3 status in the subgroup of metastases and corresponding primary colorectal tumors (*n* = 22; HER-2: *p* = 0.48, HER-3: *p* = 0.06)

Metastases				
	HER-2 negative (0/1+/2-; ISH-)	HER-2 positive (2+; ISH+/3+)	HER-3 low expression (IHC score 0/1)	HER-3 high expression (IHC score 2/3)
**Primary tumors**				
**HER-2 negative** (0/1+/2-; ISH-)	16 (73%)	2 (9%)	-	-
**HER-2 positive** (2+; ISH+/3+)	4 (18%)	0 (0%)	-	-
**HER-3 low** expression (IHC score 0/1)	-	-	3 (14%)	3 (14%)
**HER-3 high** expression (IHC score 2/3)	-	-	2 (9%)	14 (64%)

### HER-3 expression in liver metastases and corresponding primary tumors

Overall, HER-3 expression was assessed for 208 tissue specimens of liver metastases and 22 of primary tumors. We found high expression (IHC 2+ and IHC 3+) in 75.0% samples (*n* = 156) of CRLM and in 72.7% of the primary tumors (*n* = 16) (Table 3). Heterogeneity or focal expression of HER-3 was detected in 75% tissue samples comparable to HER-2 expression analyses. HER-3 expression in metastases did correlate with the HER-3 expression in corresponding primary tumors only in trend (*p* = 0.06). About 64% (*n* = 14) had high HER-3 expression in both tissue samples, the primary and the metastases (Table 4).

Similar to the results of HER-2 expression analysis, HER-3 was found in a greater extend in metachronous metastases (*n* = 72; 80%) and in metastases of colon cancer (*n* = 52; 78.8%).

### Correlation of HER-2 and HER-3 expression with clinical parameters and outcome

HER-2 and HER-3 expression analyses were correlated with clinico-pathological parameters (such as gender, primary tumor localization, UICC status at diagnosis), OS and CSS and were tested for statistical significance. These results are presented in Table 1 and Table 2. We did not find any correlation between high expression of HER-2 or HER-3 and clinico-pathological parameters. With a mean follow up time of 56.7 months, patients with high HER-3 expression showed a better CSS (*p* = 0.037) and a better OS (*p* = 0.049) (Figure 3). The 5-year CSS of patients with high HER-3 expression was 43.5%, whereas patients with low HER-3 expression had a 5-year CSS of 23.9%. The 10-year survival (CSS) was 26.1% (high HER-3 expression) versus 15.9% (low HER-3 expression). Analyses of the prognostic significance of HER-2 expression revealed no difference in CSS (*p* = 0.963) or OS (*p* = 0.747) (Figure 4).

## DISCUSSION

HER-2 and HER-3 are members of the EGFR superfamily and involved in central molecular pathways (PI3K/AKT and MAPK) in pathogenesis of solid tumors [32]. HER-2 dimerizes preferentially with the HER-3 receptor initiating potential signal transduction leading to cell proliferation, angiogenesis and formation of metastases [33]. Several studies have reported varying expression levels of HER-2 and HER-3 and evaluated their correlation with clinico-pathological parameters and prognosis but with divergent results [23][28][29][34][35]. Still, in a variety of solid tumors, the HER-2 receptor represents an effective target for monoclonal antibodies such as trastuzumab being approved for metastatic gastric cancer, pertuzumab [36] or antibody-drug-conjugates such as T-DM-1 [37] and for small tyrosine kinase inhibitors like lapatinib [38] and afatinib [39]. Besides, also HER-3 is considered as an important therapeutic target. Anti-HER-3 antibodies such as MM-121 (http://ClinicalTrials.gov: NCT01451632), RG7116 (http://ClinicalTrials.gov: NCT01482377) and U3-1287 [40] are currently being tested in several clinical trials spanning various patient populations including CRC patients. In metastatic CRC, data about significance of HER-2 and HER-3 overexpression is lacking. Therefore, we aimed to evaluate the expression pattern of HER-2 as well as HER-3 in CRLM to explore their possible prognostic values and verify if they could serve as potential targets in patients with metastatic CRC.

In the present analysis, high HER-3 expression was detected in 75% of CRLM and in 73% of resection specimens of primary tumors in CRC patients. Even though several studies assessed cytoplasmatic [41][42][43] rather than membranous staining pattern for the HER-3 receptor, we considered membranous immunostaining of the tumor cells only according to manufacturer's instructions. HER-3 stained tissue samples were similar to the HER-2 staining pattern and were characterized by distinct heterogeneity or focal expression (75% of tissue specimens had ≤30% of HER-2/HER-3 positive cancer cells) as observed in previously published studies in gastrointestinal cancer [20][23]. The frequency of exclusive membrane staining of HER-3 were also reported in studies on primary tumors in CRC patients by Ledel et al. [29] (*n* = 236) and in colon cancer patients by Beji et al. [34] (*n* = 110). Similar to our data, Ledel et al. [29] showed a high HER-3 expression in 70% of primary tumors and in 75% in corresponding CRC lymph node metastases. In previous studies the HER-3 expression had prognostic implications however with ambiguous findings regarding the clinical outcome [29][34][44][45].

In line with several studies of CRC patients [26][27], we did not find a prognostic benefit for patients with a positive HER-2 status or any correlation between positive HER-2 status and clinico-pathological parameters. As demonstrated in our preliminary studies [23], high HER-2 expression was found mainly in primary tumors in rectal cancer patients (*n* = 4; 18.2%). The rate of HER-2 positivity in metastases was 8% analogous to findings from Aprile et al.[28] (*n* = 50) with HER-2 overexpression in 12% of brain metastases. Furthermore, similarly high levels of concordance were reported for metastases and corresponding primary tumors in CRC patients [28][46]. However, the change from positive HER-2 status in primary tumors to HER-2 negative status in metastases was detected in about 20% (*n* = 4) of cases. Although this result is limited due to the small amount of assessed primary tumors, this down-regulation of HER-2 might be induced by multimodal treatment, as described for breast cancer [47][48] or is an effect of metastatic progression. Therefore, representative slides of the resected tumor rather than metastases may be adequate to evaluate the HER-2 status as a potential target for a specific treatment. Nevertheless, targeting HER-2, HER-3 and other EGFR members simultaneously could have useful applications in metastastic CRC treatment. Although trastuzumab is approved for HER-2 positive metastatic gastric cancer, clinical trials on trastuzumab therapy for (metastatic) CRC are still lacking. In CRC, data reported that a HER-2 overexpression is also involved in response to anti-EGFR therapies. Bertotti et al. [49] identified *in vitro* the HER-2 overexpression and gene amplification to be a negative determinant in addition to KRAS mutation for response to an anti-EGFR therapy like cetuximab in metastatic CRC. Dual inhibition with anti-EGFR antibodies (cetuximab or pertuzumab) and anti-HER-2 antibody (tyrosine kinase inhibitor lapatinib) led in preclinical studies to a significant regression of the tumor (−75%) in comparison to treatment with cetuximab or lapatinib alone. Further analyses and clinical phase II studies are also consistent with these results (DUX study) [50][51] and showed increased survival and response rates and even doubled progression-free time with dual inhibition versus single antibody therapy.

In cancers with ligand-dependent activation of HER-3, several studies suggest therapeutic potential of anti-HER-3 substances [31][43][52]. Scartozzi et al. [43] showed that HER-3 proved to be a predictive factor for clinical outcome in wild-type KRAS CRC patients treated with cetuximab. Currently, a clinical study evaluates treatment with MM-121 plus cetuximab versus MM-121 in combination with cetuximab plus irinotecan in CRC (http://ClinicalTrials.gov: NCT01451632). Another multicenter study is recruiting participants to evaluate RG7116 alone (part A) or in combination with cetuximab (part B) or erlotinib (part C) in patients with metastatic and/or locally advanced malignant HER-3 positive solid tumors (http://ClinicalTrials.gov: NCT01482377). The results remain to be awaited.

In conclusion, HER-2 is detectable in a relevant proportion and HER-3 is highly overexpressed in liver metastases of patients with CRC (HER-2 8%; HER-3 75%). In patients with overexpression of HER-2 and/or HER-3 with tumor progression and metastatic spread, targeting these two receptors may hypothetically be beneficial. The HERACLES phase II trial demonstrated with first results that the combination of trastuzumab and lapatinib was effective in standard therapy refractory mCRCs with HER-2 amplification [53]. Prospective clinical trials will be necessary to validate HER-2 and HER-3 as potential targets for precision medicine in the treatment of (metastatic) CRC.

## PATIENTS AND METHODS

This monocentric study included 208 patients (median age: 67.5 years; 71 female (34%) and 137 male (66%)) with CRLM, treated between April 1982 and January 2013 at the Department of General, Visceral and Pediatric Surgery, University Medical Center Göttingen, Germany. The patients' demographics and clinical characteristics are summarized in Table 1. Patients were eligible if they had CRC along with the occurrence of either synchronous or metachronous liver metastases. Additional screening tests (e. g. CT scan of the abdomen/thorax/brain, bone scintigraphy) were performed to detect signs of extrahepatic tumor manifestations and to verify if CRLM were resectable.

Distant metastases were mainly localized exclusively in the liver (*n* = 117; 56%), followed by metastases detected simultaneously in various organ systems (liver, lungs, peritoneal, bones and/or brain; *n* = 91, 44%). Most patients presented with lesions in one lobe (*n* = 105; 50%) or in two lobes of the liver (*n* = 89; 43%). A total amount of 118 patients (57%) were diagnosed UICC (Union International Contre le Cancer) stage IV with synchronous metastases (Table 1), while in 90 patients (43%) distant metastases occurred within further course of the disease (metachronous metastases). Mostly the primary tumor was located in the rectum (*n* = 142; 68%). In 66 patients (32%) it was localized in the colon. Overall, about 98% of the primary tumors were surgically removed. Only 5 patients (2%) had surgery in palliative intention due to an extraorgan extension of CRC and extended findings intraoperatively.

### Treatment - surgery and chemotherapy

In this study, 179 (86%) patients underwent liver surgery including major (*n* = 102; 57%) or minor (*n* = 77; 43%) resection of the liver for resectable metastases (Supplementary Table 1). Resectability was assessed by experienced liver surgeons and was determined by the number of metastases, size and account of lesions, the patients' constitution and comorbidity and risk of further damage with fatal outcome (e.g. hepatitis, hepatic failure). Major surgery involved resection of four or more liver segments such as (extended) hemihepatectomy whereas in minor surgery only one or up to three liver segments were resected.

Perioperative combination CTx protocols varied in accordance to patients' tumor genetic profile such as KRAS-status. Patients with a KRAS wild-typ status received the monoclonal antibody cetuximab simultaneously to CTx protocols with doublets (*n* = 25; 12%; Supplementary Table 1). Bevacizumab, a humanized monoclonal antibody against vascular endothelial growth factor (VEGF), was applied to 24 patients (12%). In 6 patients (3%), the application of cetuximab additionally to bevacizumab had been performed. One patient received an additional third antibody panitumumab, a humanized monoclonal antibody against epidermal growth factor (EGF).

### Immunhistochemistry protocol

The HER-2 status and HER-3 expression level were determined in 208 formalin-fixed paraffin-embedded tissue samples from hepatic CRC metastases. In addition 22 HER-2 stained resection specimens could also be included in this study, so that 22 matched pairs of CRLM samples and corresponding primary cancer resection specimens were available for further analyses.

HER-2 immunostaining was conducted using a PATHWAY® anti-HER-2/neu (4B5) rabbit monoclonal antibody (Ventana Medical Systems, Mannheim, Germany) on a Ventana BenchMark XT immunostainer (Ventana, Tucson, AZ, US) and visualized by the ultraView Universal DAB Detection Kit (Ventana Medical Systems, Mannheim, Germany). HER-3 expression was determined by using the anti c-erbB-3/HER-3 rabbit monoclonal antibody (clone SP71; Spring Bioscience, Pleasanton, USA). Examples of the immunohistological staining of HER-3 are shown in Figure 2.

For the evaluation of HER-2 and HER-3 we used the testing protocol, which had been developed within the ToGA-trial and is now well established to determine HER-2 expression and amplification in patients with adenocarcinomas of the stomach and the gastroesophageal junction [20][54] and which was used in primary rectal adenocarcinomas before [23].

**Figure 1:**
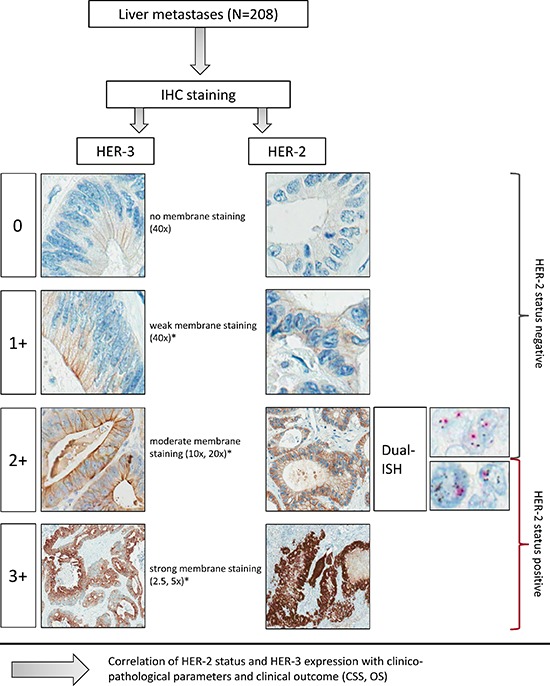
Examples of HER-2 and HER-3 immunohistochemical staining and Dual-ISH according to the HER-2 algorithm This figure shows the HER-2 algorithm with examples of HER-2 and HER-3 according to the magnification rule as adopted from gastric cancer scoring for HER-2. *in ≥10% tumor cells in resection specimens.

Scoring criteria included the magnification rule, which allowed an objective and standardized evaluation (Figure 1). The stained tissue samples were classified according to their staining intensity regarding certain magnification in the light microscope. Tumor cells were assessed positive if they had only distinct, regularly striped intercellular membrane staining. The circularity of membrane staining was not a prerequisite for a positive membrane staining. Due to a lack of luminal receptors situated in intestinal glands basolateral or lateral membrane stainings were also taken into account. Cytoplasmic, nuclear or granular staining that may occur among other cases of intestinal metaplasia was not included [25].

**Figure 2:**
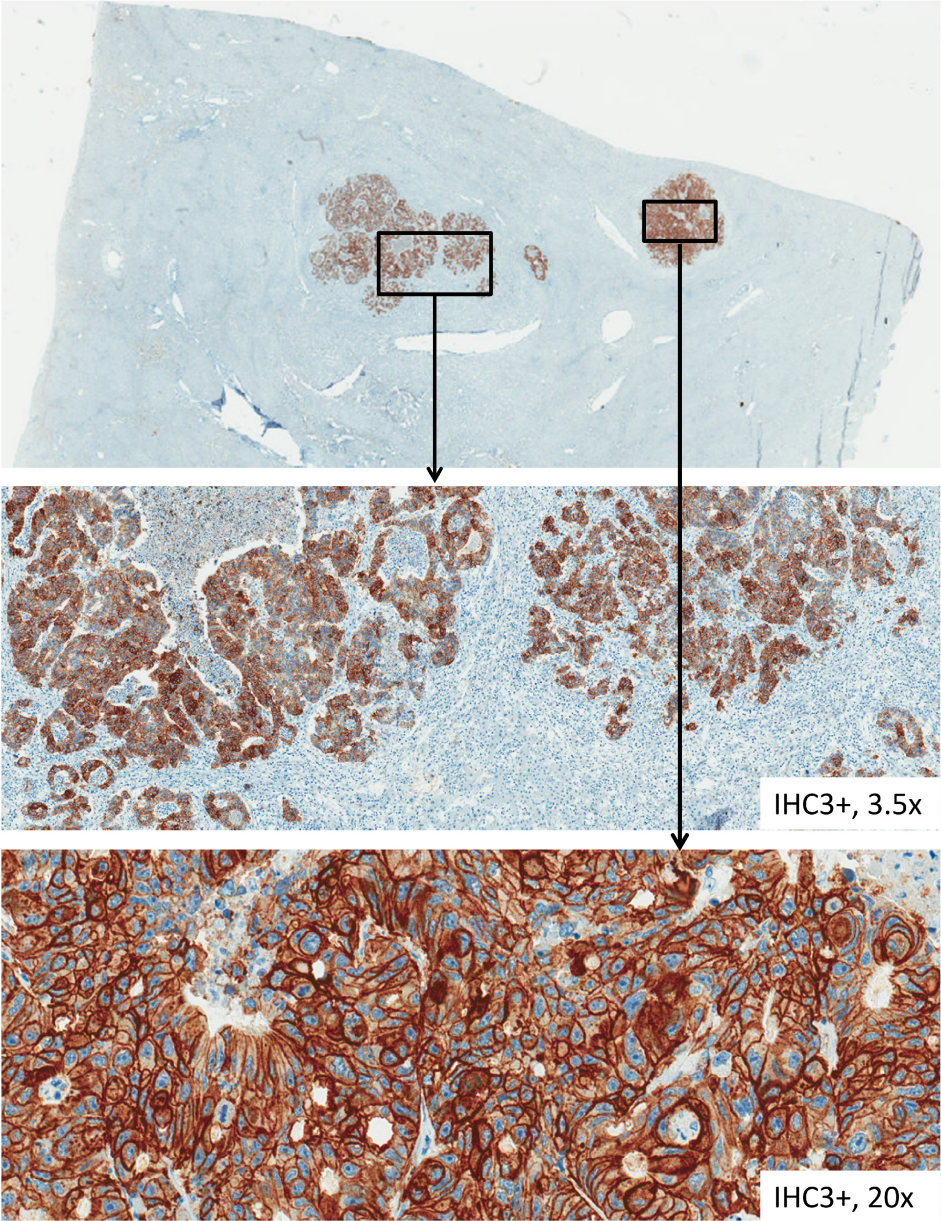
HER-3 immunohistochemical staining of tissue samples from liver metastases This figure pictures tissue samples from HER-3 immunohistochemical staining from CRC liver metastases with various factors of magnification.

**Figure 3:**
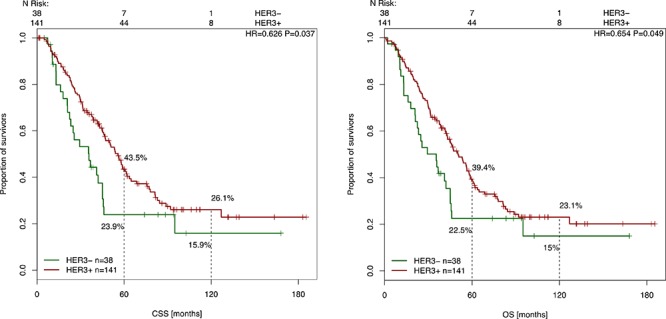
Cancer-specific and overall survival after liver surgery in correlation with HER-3 expression Kaplan-Meier curve for CSS and OS of CRC patients with high and low HER-3 expression levels (*p* = 0.037, *p* = 0.049). The Cox model for CSS and OS based on HER-3 status from 179 resection specimens of liver metastases.

**Figure 4:**
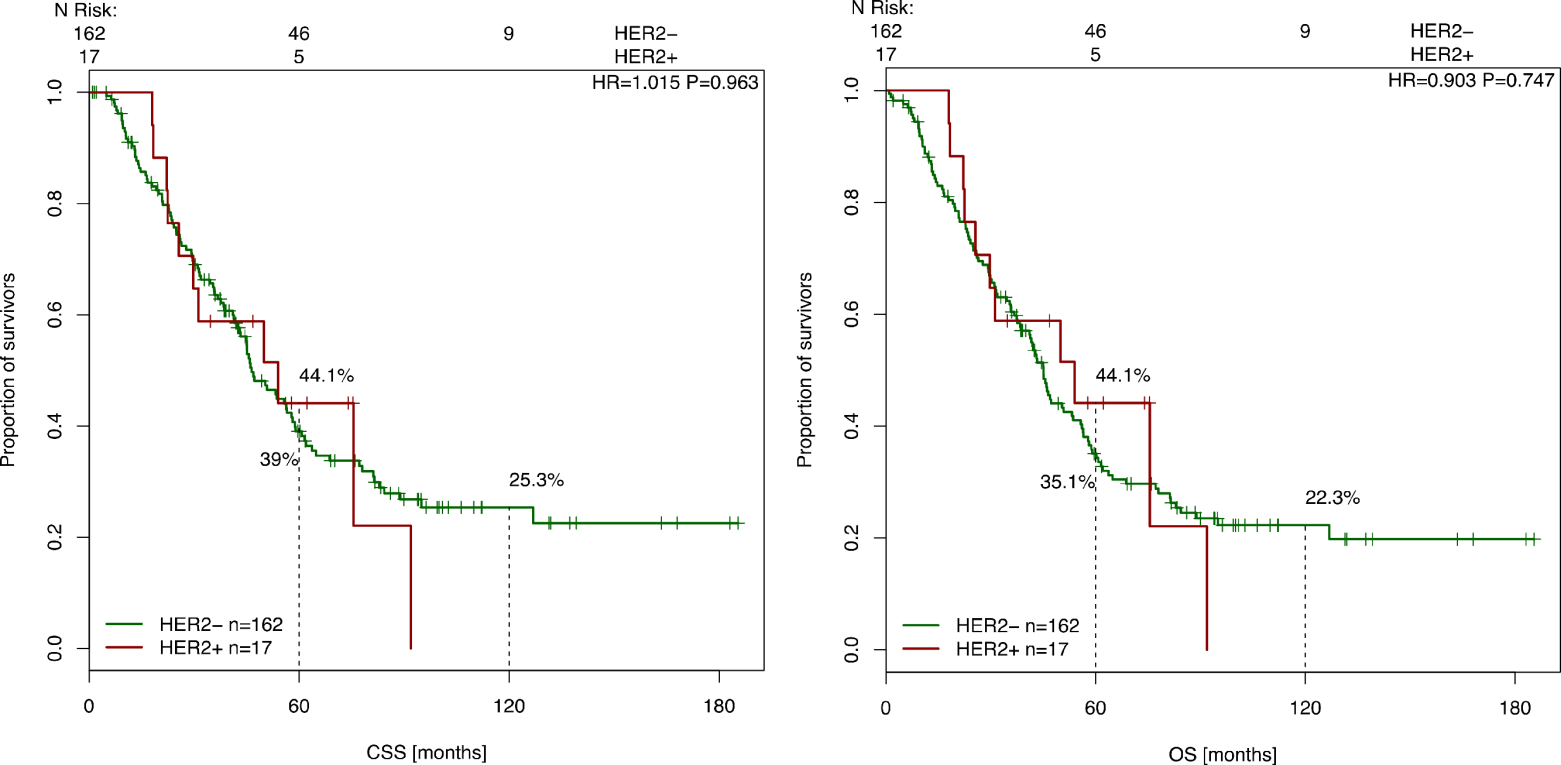
Cancer-specific and overall survival survival after liver surgery in correlation with HER-2 expression Kaplan-Meier curve for CSS and OS of CRC patients with high and low HER-2 expression levels (*p* = 0.963, *p* = 0.747). The Cox model for CSS based on HER-2 status from 179 resection specimens of liver metastases.

Tumor samples were considered IHC 2+ or IHC 3+, if at least 10% of the tumor cells had medium (10x, 20x magnified, IHC 2+) or strong membrane staining at low magnification (2.5x, 5x, IHC 3+). No membrane staining was scored IHC 0 and weak membrane staining in at least 10% of the tumor cells was defined as IHC 1+ (40x magnified).

HER-2 samples scored IHC 2+ were further prepared for detection of gene amplification using Dual-in-situ-hybridization (Dual-ISH). In HER-3 stained samples no assessment of gene amplification had been performed (Figure 1).

### Dual-ISH protocol

Dual-ISH was performed in HER-2 cases with equivocal membrane staining (if scored IHC 2+) according to manufactures recommendations by using the Ventana INFORM HER-2 Dual ISH/DNA Probe Cocktail and visualized utilizing two-color chromogenic *in situ* hybridization (ultraVIEW SISH Detection KIT and ultraVIEW Red ISH DIG Detection Kit, Ventana Medical Systems, Mannheim, Germany). HER-2 gene amplification was determined by the count of visualized copies of the HER-2 gene and visualized copies of chromosome 17. Ratios above 2.0 were considered amplified. IHC 3+ or IHC 2+/Dual-ISH positive (amplified) were classified HER-2 positive; IHC 0, IHC 1+ or IHC 2+/Dual-ISH negative (not amplified) were defined HER-2 negative [23]. IHC and Dual-ISH analyses were performed independently by two different observers (HS and IN).

### Statistical analysis

The association of HER-2 and HER-3 expression levels with other clinico-pathological parameters was assessed using Fisher's exact test. Survival rates were supplied by means of Kaplan-Meier analysis and tested using the Cox proportional hazards model. Overall survival (OS) was defined as interval between surgery of the metastasis and death of any cause and cancer-specific survival (CSS) was defined as time from surgery of the metastasis and cancer related death. The *p*-value was set to *p* < 0.05 to be considered statistically significant. Statistical analyses were conducted using *R* statistical computing environment version 3.1.1 [55]. Survival analysis was performed using the R package *survival*.

## SUPPLEMENTARY TABLE


