# Supplementary material for: HER-2 and HER-3 expression in liver metastases of patients with colorectal cancer
Source: Oncotarget. 2015 Apr 13;6(17):15065–76. doi: 10.18632/oncotarget.3527 (PMC4558136; doi:10.18632/oncotarget.3527)
Supplement: Supplementary file 1 [file oncotarget-06-15065-s001.pdf]

SUPPLEMENTARY TABLE

Supplementary Table S1: Systemic Treatment

Treatment	*N* = 208	%
**Surgery**		
Yes	179	86
No (unresectable)	29	14
**Surgical procedure** (*n* = 179)		
Major liver surgery	102	57
Minor liver surgery	77	43
**Synchron metastases (*n* = 118)**		
**Chemotherapy**		
5-FU mono	29	25
5-FU + oxaliplatin	61	52
5-FU + irinotecan	15	13
No chemotherapy	13	11
**Second-line chemotherapy**		
5-FU mono	3	3
5-FU + oxaliplatin	11	9
5-FU + irinotecan	27	23
No second-line chemotherapy	77	65
**Metachronous metastases (*n* = 90)**		
**Chemotherapy**		
5-FU mono	11	12
5-FU + oxaliplatin	25	28
5-FU + irinotecan	15	17
No chemotherapy	39	43
**Second-line chemotherapy**		
5-FU mono	0	-
5-FU + oxaliplatin	8	9
5-FU + irinotecan	6	7
No second-line chemotherapy	76	84
**Biological agents**		
Cetuximab	25	12
Bevacizumab	24	12
Cetuximab + bevacizumab	6	3
Cetuximab + bevacizumab + panitumumab	1	0.05
None	152	73
